# Design, implementation, and inferential issues associated with clinical trials that rely on data in electronic medical records: a narrative review

**DOI:** 10.1186/s12874-023-02102-4

**Published:** 2023-11-16

**Authors:** Alan Leviton, Tobias Loddenkemper

**Affiliations:** Department of Neurology, Boston Children’s Hospital, Harvard Medical School, Boston, MA USA

**Keywords:** Electronic health record, Learning health system, Treatment outcome, Common data elements, Ethics, Data accuracy, Government regulation

## Abstract

Real world evidence is now accepted by authorities charged with assessing the benefits and harms of new therapies. Clinical trials based on real world evidence are much less expensive than randomized clinical trials that do not rely on “real world evidence” such as contained in electronic health records (EHR). Consequently, we can expect an increase in the number of reports of these types of trials, which we identify here as ‘EHR-sourced trials.’ ‘In this selected literature review, we discuss the various designs and the ethical issues they raise. EHR-sourced trials have the potential to improve/increase common data elements and other aspects of the EHR and related systems. Caution is advised, however, in drawing causal inferences about the relationships among EHR variables. Nevertheless, we anticipate that EHR-CTs will play a central role in answering research and regulatory questions.

## Introduction

Some who study randomized clinical trials broadly dichotomize them into explanatory (does this treatment work under ideal (or what Schwartz and Lellouch identified as “laboratory” conditions) [1] and efficacious (does this treatment work in the real world.

(what Schwartz and Lellouch identified as “normal” conditions) [2], while others prefer to see them as a continuum along this scale [1, 3, 4]. The ideal conditions needed for explanatory trials, however, are expensive, and do not allow inferences about generalizability of the findings. On the other hand, the normal conditions providing real world data are inexpensive, and do allow inferences about generalizability of the findings [5]. During the half-century since the ‘real world’ concept was introduced, 38 definitions of real-word data (RWD) have been offered with most approximating “data collected in a non-randomized controlled trial setting” [6].

Multiple forces are contributing to a rapid expansion of interest in real world data for clinical trials. These forces include the increasing availability of electronic data and the acceptability of real world evidence in support of applications for approval of medical products for marketing. We begin this selected review of the literature with a description of these recent developments driving the focus on this topic. We follow this with a discussion of the designs of studies intended to evaluate therapies and interventions using real world evidence, including the advantages and limitations of each, the inferences that can be drawn from study results, and issues related to recruiting/enrollment and ethics. We conclude with comments about the future.

### Increasing availability of electronic data

The increasing availability of large integrated data sources [7–9] bodes well for clinical trials that rely on real world data, including the contents of the electronic health record. So does the availability of “learning health systems that use routine data from service delivery and patient care to generate knowledge to continuously improve healthcare” [10]. Groups of learning health systems are coming together to form networks, such as the nine Clinical Research Networks and the two Health Plan Research Networks that are part of Cornet [11]. The creation of an electronic health record data aggregation platform by one of the large vendors is likely to result in more and/or larger networks that will be able to function as integrated learning health systems [12].

More recently, the United States Centers for Medicare & Medicaid Services proposed rule (CMS 0057-P) is intended to advance interoperability among health information exchanges (entities such as hospitals, and public health agencies in states or regions that help share information among groups that have a legitimate need for it) [13]. To exchange data with health information exchanges, hospitals and networks of hospitals are expected to use application programming interfaces that can gather information from multiple sources and aggregate it in one place [14]. This would allow access specific pieces of information, rather than having to sort through pages of unnecessary or unhelpful records. The likely result is expected to be many more clinical trials based on real world data than in the recent past.

### Government encouragement

The European Medicines Agency, the United States Food and Drug Administration, and Health Canada now accept real world evidence (RWE) in applications for approval of medical products for marketing [15].

The Food and Drug Administration defines RWD as “data relating to patient health status and/or the delivery of health care routinely collected from a variety of sources” and defines RWE as “clinical evidence about the usage and potential benefits or risks of a medical product derived from analysis of RWD,” regardless of the type of study design [16]. In the announced framework for its RWE program for drug and biological products, Food and Drug Administration acknowledged that clinical trials that use EHR data are acceptable, as are administrative claims data, or registry data [17],

The perceived need for transparency in applications to FDA prompted one group to create “The Structured Pre-Approval and post-approval Comparative study design framework to generate valid and transparent real-world Evidence (SPACE)” for identifying design elements, feasibility and validity concerns, and for documenting decisions [18]. An extension to SPACE, identified with another acronym, SPIFD for “Structured Process to Identify Fit-For-Purpose Data tool,” provides a guide to identify fit-for-purpose data required for the Food and Drug Administration’s (FDA) real-world-evidence (RWE) program [19].

Increasingly, routinely collected data are seen as an attractive source for post-marketing surveillance, complementing established spontaneous report mechanism [20, 21].

Here we focus on clinical trials that rely on data provided by EHRs, and what others have come to identify as "EHR-sourced" trials [22].

### Designs: clinical effectiveness and implementation (Table 1)

**Table 1:** Design characteristics of clinical trials (Ideal Types)

	**Clinical effectiveness**	**Implementation**
Unit studied	Patient	Provider (clinical unit/system)
Intervention	“clinical” intervention (e.g., education, reminders, guidance)	“clinical” intervention (e.g., education, reminders, guidance)
Outcomes	Health outcomes (e.g., medication adherence, missed appts, cost reduction)	Adoption/uptake of intervention

EHR-sourced trials can be evaluated along a spectrum anchored at one end by those intended to assess only the clinical effectiveness of the intervention and anchored at the other end by those that assess only the adoption/uptake/acceptance of the intervention by groups of providers and institutions.

Increasingly, the word ‘hybrid’ is associated with EHR-sourced trials that try to evaluate (to varying degrees) both clinical effectiveness and acceptance of the intervention [23–25]. The three types of hybrid designs are:

- Type 1: tests the clinical effectiveness of the intervention (reducing elevated blood pressure or blood glucose level) while observing and gathering information on the implementation,
- Type 2: dual testing of clinical effectiveness and implementation interventions/strategies, and
- Type 3: evaluates the adoption of an implementation strategy while observing and gathering information on its clinical effectiveness.

In the absence of obvious boundaries, some investigators avoid use of these labels (at least in the title). Only one report applied the term ‘continuum’ to the spectrum along the Type 1 to Type 3 classification [26]. More information about implementation strategies is available elsewhere [27, 28], as are examples of implementing self-management strategies for people with diabetes [29, 30], with asthma [31, 32], with hypertension [33, 34], and with epilepsy [35–38].

### Justification for EHR based clinical trials

“*The often stringent inclusion and exclusion criteria destined to provide for homogeneous study populations reduce the generalizability of randomized clinical trial results*” [39]. Findings reported from observational studies, have, on important occasions, not been confirmed in randomized clinical trials [40–53]. This has been emphasized with the finding that in one-sixth of all comparisons between randomized clinical trials and observational studies, “there was a significant difference and the estimates pointed in opposite directions” [54].

The findings of EHR-sourced trials, especially those conducted in populations that are likely to benefit from the results of the study, tend to be more informative about what works in the real world than are the findings of explanatory trials (RCTs) [40, 55]. EHR-sourced trials are most suited to answer the question, “Will this intervention work in this population?” Investigators are encouraged to ‘embed’ randomization in learning health systems and networks [56].

### Designs: randomized trials

The randomized trial design of an EHR-sourced trial can be identical to that of a randomized clinical trial in the most restrictive set of subjects. Two of the more common modifications of the conventional randomized clinical trial are conventional cluster randomized design and stepped-wedge cluster randomized design.

**Cluster randomized trials** compare the results of different interventions among groups of people whose members have an identifiable feature in common [57, 58]. They are most appropriate when.

- the intervention evaluated is likely to be implemented subsequently among other patients who have the randomized groups’ characteristics.
- the intervention carries a high risk of “contamination” (*i.e.*, individuals randomized to different comparison groups are in frequent contact with one another and thus may be influenced (‘contaminated’) by recipients of the alternative treatment).
- they have practical advantages over individual randomization (because of lower implementation costs, or administrative convenience).

Cluster randomized trials, however, pose potential ethical concerns (*v.i.*, Ethics section).

In a stepped wedge design, an intervention, an intervention is rolled out sequentially to the trial participants (either as individuals or clusters of individuals) [59, 60]. The order in which the different individuals or clusters receive the intervention is determined at random and, by the end of the random allocation, all individuals or groups will have received the intervention.

Among the reasons investigators chose the stepped wedge design in preference to others are:

- The stepped wedge design avoids the logistical barriers that accompany efforts to implement the intervention simultaneously in many clusters,
- a lack of equipoise for the intervention made the investigators feel it would be unethical to deny the intervention to some groups,
- a desire to avoid the ‘disappointment effects’ possible in a parallel trial that follow from colleagues at some clusters who decide to drop out of the study when randomised to the control arm,
- higher statistical power associated with clusters functioning as their own controls
- the ability to adjust for time trends in outcomes,.
- logistical, practical, or financial constraints require that the intervention be implemented in stages [61–63].

In such circumstances, determining the order in which participants receive the intervention at random is likely to be both morally and politically acceptable and may also be beneficial for trial recruitment.

The step wedge design was chosen for a study that evaluated if the presence of a seizure dog in the home reduced seizure and injury frequency [64]. “This design was chosen because it allows for rollout of the intervention to all participants. … The current capacity of the assistance dog schools participating in the EPISODE study would not permit simultaneous rollout of the required number of seizure dogs to all participants.” In addition, “blinding of the participants would be impossible.”

### Group sequential design

Some patients exposed to an intervention that might be a source of potential benefit or harm can be monitored sequentially for either of these possibilities [65]. Group sequential design, also known as interim analysis offers an opportunity to make decisions along the way about whether or not future patients should be so exposed [66, 67].

### Inferences

Randomized clinical trials (RCTs) are considered the "gold standard" for evaluating the safety and efficacy of new therapeutic agents because of their high quality data and strict inclusion and exclusion criteria [68, 69]. In contrast, however, EHR-sourced trials are fraught with potential biases and limited quality of the data in the EHRs [70–79]. These biases and limitations include selection bias [80–83], protopathic bias, [84–86], missingness and other data quality limitations, [78, 87–94], time-orientation challenges [88, 95], and potential confounding [96, 97]. These biases and limitations have the potential to limit severely the causal inferences that can be drawn from the contents of EHRs [98–103].

Although efforts are underway to address some of them, structural limitations, such as the paucity of common data elements recommended for research purposes by the National Institutes of Health [104] will limit how much bias can be reduced soon [77, 91, 105–113]. Nevertheless, planned EHR-sourced trials of technological care advances and changes in practice have the potential to enhance data quality in the EHR by requiring participating organizations to include selected common data elements, or by providing other data details (*i.e.*, granularity) not previously included in the EHR. Data quality is also likely to be improved by the apparently increasing use of home monitoring devices, wearable devices, templated smartforms for documentation, automated transcription, artificial intelligence and natural language processing (to extract salient information from the EHR) [114, 115].

The Cochrane risk-of-bias groups have created tools for assessing “risk of bias in non-randomized studies of interventions” (ROBINS-I) [116], assessing risk of bias in randomised trials (RoB 2) [117], and even in cluster-randomized trials [118], as well as assessing risk of missing evidence (ROB-ME) [119, 120].

### Recruiting/enrollment

Learning healthcare systems, with their large collections of EHRs, are likely to include a broad patient population with characteristics as close as possible to patients in routine clinical practice, whose responses to interventions will maximize the generalizability and applicability of trial results [121]. Data about such patients can help design suitable EHR-sourced trials [122]. EHR query tools can identify potential candidates for these clinical trials when the selection criteria are expressed in structured digital format [123, 124]. Once identified, eligible subjects can be enrolled electronically, either by email or via the patient portal [125, 126], which is recommended because of its security [127]. Doing so, however, will not eliminate bias [127–131].

Because recruitment for a randomized clinical trial can take a long time, alternative arrangements have been sought. One option is the ‘trial within cohorts’ design, which uses the infrastructure of an observational cohort study to identify possible participants for a randomized trial [132]. Upon cohort enrollment, all participants provide consent for being randomized in future studies without being informed. When a new treatment becomes available for evaluation, those randomized to the treatment arm are offered the new treatment, which they can choose to refuse. Those randomized to the standard of care arm are not informed about the trial and continue to receive standard of care as part of the cohort study. Patients do not appear to have ethical objections to serve as control without further notice [133, 134].

### Ethics

The main ethical issue associated with EHR-sourced trials is when and how consent is obtained. Of 1988 EHR-sourced trial reports published during the years 2014 to 2019, 7% did not include a statement about participant consent and only 7.0% reported a waiver [135]. Cluster randomization studies were more likely than others to obtain consent or provide details about consent.

#### The role of institutional review boards

Given their complex coordination across multiple sites, EHR-sourced trials, in general, pose a challenge for research oversight mechanisms, including ethical review by multiple institutional review boards [136]. The Common Rule regulations allow waiver of consent by an institutional review board when the research poses no more than minimal risk, doing so does not adversely affect the rights or welfare of the participant, and obtaining direct consent is impracticable [137]. When a waiver is granted, subjects can/should still be informed of the nature of the study and how it might affect them, and perhaps even offered an opt-out option [138]. Minimal-risk determinations are the provenance of each institutional review board [139], but considerations can/should include “clinical equipoise, practice variation, research methods such as cluster randomization, and patients' perspectives” [140]. Yes, patients’ perspective! In one survey, three quarters of subjects approved obtaining post-randomization the consent of only those assigned to the treatment arm [141].

Nevertheless, suggested options “to make sure” everyone is on board, include investigators consulting with the institutional review board, [142] obtaining written informed consent for all participants before randomization, [143] and opportunities to ‘opt-out’ [144–146]. More than two-thirds of a national sample of US adults “reported interest in notification of research using their identified health information, and 40% reported interest in notification if the health information was deidentified [147].

#### Cluster randomized studies

In cluster randomized trials the unit of randomization might be a hospital or network of hospitals that might ask physicians to treat all patients with disease X alike and collect data from patients. The benchmark ethical recommendations about who needs to consent are in “The Ottawa statement on the ethical design and conduct of cluster randomized trials” [148] and in the 2016 Council for International Organizations of Medical Sciences (CIOMS) ethical guidelines [149]. Additional guidance has come from the FDA [150] and from “a guide for the perplexed” [151].

#### Stepped-wedge cluster randomized studies

Stepped-wedge cluster randomized trial designs pose additional challenges [143]. In contrast to parallel cluster randomized trials in which clusters are randomized at the outset to either intervention or control arms, stepped-wedge cluster randomized trials offer the intervention sequentially to each cluster so that each cluster begins the trial as its own control, and then receives the intervention [59, 152, 153]. This approach is thought to avoid ethical concerns about the denial of a desired intervention to participants in control groups [154–156].

The Consolidated Standards of Reporting Trials has an extension for stepped-wedge cluster randomized trial that includes whether or not consent was obtained, the purpose of consent, when consent was sought (i.e., before or after randomization), and the forms of consent (differences between intervention and control clusters) [157]. A review of the stepped-wedge cluster randomized trials in the National Institutes of Health's Health Care Systems Research Collaboratory found that decisions to use the stepped-wedge cluster design were more often justified by practical and epistemic reasons than by ethical ones [143].

### Limitations of this report

The main limitation of this report is that it is not a comprehensive overview of the literature. We specifically chose studies to show the breadth of the perspectives they offer about the designs most suitable for using EHR data, and to emphasize the data limitations, potential biases and other challenges that might hamper drawing causal inferences based on EHR contents.

### The future

We see many phenomena contributing to large increases in the use of EHR data for clinical trials. First, the acceptance of real world evidence by authorities charged with assessing the benefits and harms of new therapies [158, 159], is highly likely to provide a strong impetus for EHR-sourced trials in preference to randomized clinical trials [160, 161]. Consequently, we can expect continued increase in the number of reports of EHR-sourced trials as well as efforts to have EHR data become “regulatory-grade) [69, 162].

Second, efforts to encourage an expanded set of common data elements for EHRs appear promising [105, 163, 164]. These efforts have the potential to lead to improvements have the potential to increase the validity of future EHR-sourced trials [165, 166].

Third, advances in artificial intelligence and machine learning bode well for significant improvement in the quality and quantity of information contained in the EHR [91, 115, 167, 168]. Although some will undoubtedly apply to natural language processing, we have little knowledge of the extraordinary achievements we can expect [169].

In light of these phenomena, EHR-sourced trials have a very bright future. Nevertheless, we are less optimistic that EHR data will be useful any time soon for providing valid information for drawing causal inferences about the relationships between EHR variables.

## Conclusions

EHR-sourced trials have conceptual and logistic properties that make them especially attractive for future studies of what does and what does not work among those with the characteristics of the participants.

## Data Availability

Data sharing not applicable to this article as no datasets were generated or analysed during the current study.
